# Hybrid Bone Transport for Large Bone Defects of the Lower Limb: A Retrospective Cohort Study of Early Clinical Outcomes From a Single Center

**DOI:** 10.7759/cureus.108585

**Published:** 2026-05-10

**Authors:** Paul Fu-Xiang Kong, Muhammad Yasir Ahmad Muslim, Nazari Ahmad Tarmuzi

**Affiliations:** 1 Orthopaedic Surgery, Sarawak General Hospital, Kuching, MYS; 2 Orthopaedic Surgery, Hospital Shah Alam, Shah Alam, MYS; 3 Orthopaedic Surgery, KPJ Kuching Specialist Hospital, Kuching, MYS

**Keywords:** critical bone defects, distraction osteogenesis, external fixation index, hybrid bone transport, ilizarov technique

## Abstract

Background

Large bone defects of the lower limb remain a reconstructive challenge. Traditional Ilizarov bone transport is effective but associated with prolonged external fixation. Hybrid techniques combining internal and external fixation have been utilized to reduce the external fixation index (EFI). This study evaluates clinical, radiological, and functional outcomes of hybrid bone transport techniques.

Methods

A retrospective cohort study was conducted at a tertiary trauma center between April 2022 and March 2026. Patients with post-traumatic lower limb bone defects treated with hybrid bone transport techniques were included. The primary outcome was the EFI. Secondary outcomes included time to union, complications, and bone functional outcomes assessed using the Association for the Study and Application of the Method of Ilizarov (ASAMI) criteria.

Results

Six patients (mean age = 32.2 years) with a mean defect size of 10.3 ± 3.6 cm were included. The mean time to union was 8.3 ± 1.8 months. The mean time on frame was 99.8 ± 30.3 days. The mean EFI was 14 ± 3.2 days/cm. All patients achieved union. Complications included malalignment requiring revision (n = 1) and joint stiffness (n = 2). The ASAMI bone and functional outcomes were good and excellent.

Conclusion

Hybrid bone transport techniques substantially reduce EFI compared to traditional Ilizarov methods while maintaining high union rates and favorable functional outcomes.

## Introduction

The management of large segmental bone defects following trauma remains one of the most challenging problems in orthopedic surgery. Established reconstructive options include vascularized bone grafting, induced membrane technique, and distraction osteogenesis via bone transport [1]. We primarily utilize the bone transport technique for large bone defects of the lower limbs due to a lack of expertise to harvest vascularized fibula grafts and prior mixed results with the induced membrane technique. Both these techniques carry significant donor site morbidity and lead to dissatisfaction [1].

Distraction osteogenesis remains the most versatile due to its ability to regenerate bone biologically without the need for grafting [2]. However, the conventional Ilizarov technique is not without its disadvantages. Among these are local complications of pin tract infection and adjacent joint stiffness, in addition to being cumbersome and requiring a prolonged period of external fixator use [3,4].

As these disadvantages prove unpopular with the modern-day patient [1,4], we have applied different hybrid techniques to reduce the external fixation index (EFI). These techniques are described below, along with a report of the clinical outcomes. This study evaluates the benefits of hybrid transport for the regional limb reconstruction community, addressing its limited adoption caused by concerns regarding cross-infection to internal implants. The primary outcome of this study is the EFI, and secondary outcomes are time to union, time on frame, complications, and the bone and functional outcomes.

## Materials and methods

Study design and setting

This was a retrospective, single-center observational study conducted at Sarawak General Hospital, a tertiary trauma referral center in Kuching, Sarawak, Malaysia. Data were collected between April 2022 and March 2026.

Patient selection

Patients were considered eligible for inclusion if they presented with or acquired a bone defect of the lower limbs from traumatic injuries and were treated with a form of hybrid transport with or without the addition of the induced membrane technique (IMT). Patients with a history of osteomyelitis or fracture-related infections were excluded. Included patients had at least one year of follow-up after achieving bone union and returning to function.

Cases and surgical procedures

All surgeries in this series were performed by the same surgical team. Six patients were included based on eligibility. Open fractures were debrided and temporized with spanning external fixators, and articular fractures were reconstructed and fixed. In most cases, second-look debridement was performed, followed by a rigorous preoperative planning process to decide on an optimal treatment plan. For bone loss exceeding 10 cm, the IMT was incorporated into one segment to reduce transport distance and time of frame (TOF). A combination of techniques using the principles of distraction osteogenesis was employed to achieve this goal, tailored to each specific patient. In all cases, a latency period of 10 days was observed, followed by distraction at a rate of 1 mm/day at a rhythm of four times per day.

**Figure 1:**
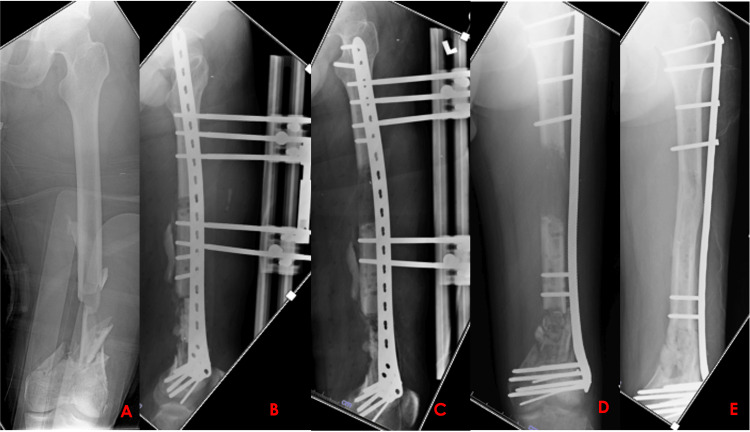
Serial radiographs of a case of acute shortening, followed by bone lengthening over plate + induced membrane technique (IMT). (A) Initial trauma film depicting injury with bone loss of 13 cm. (B) The defect is shortened by half, followed by fixation with a locking plate and application of monolateral rail and proximal corticotomy for lengthening. (C) Proximal locking screws are inserted after completion of lengthening. (D) Autologous bone graft performed for the distal IMT segment. (E) Radiograph at nine months showing fracture site union and transport segment consolidation.

In cases 1 to 3, we decided to acutely shorten (AS) half the defect (e.g., from 13 cm to 6.5 cm) during the definitive fixation, whereupon the distal segment was treated with the IMT, and the proximal segment was re-lengthened via bone lengthening (BL) over plate (BLOP) utilizing a limb reconstruction system (LRS) rail (Figure 1). Upon achieving the target length, the bone segments were locked to the plate, the LRS rail was removed, and an iliac crest autograft was performed for the distal segment.

For case 4, the defect of the tibia diaphysis was acutely docked (AD), followed by proximal segment re-lengthening as per BLOP with the LRS rail. The bone segments were locked to the plate following completion of lengthening, and the rail was removed. Further procedures to the docking site were not required.

**Figure 2:**
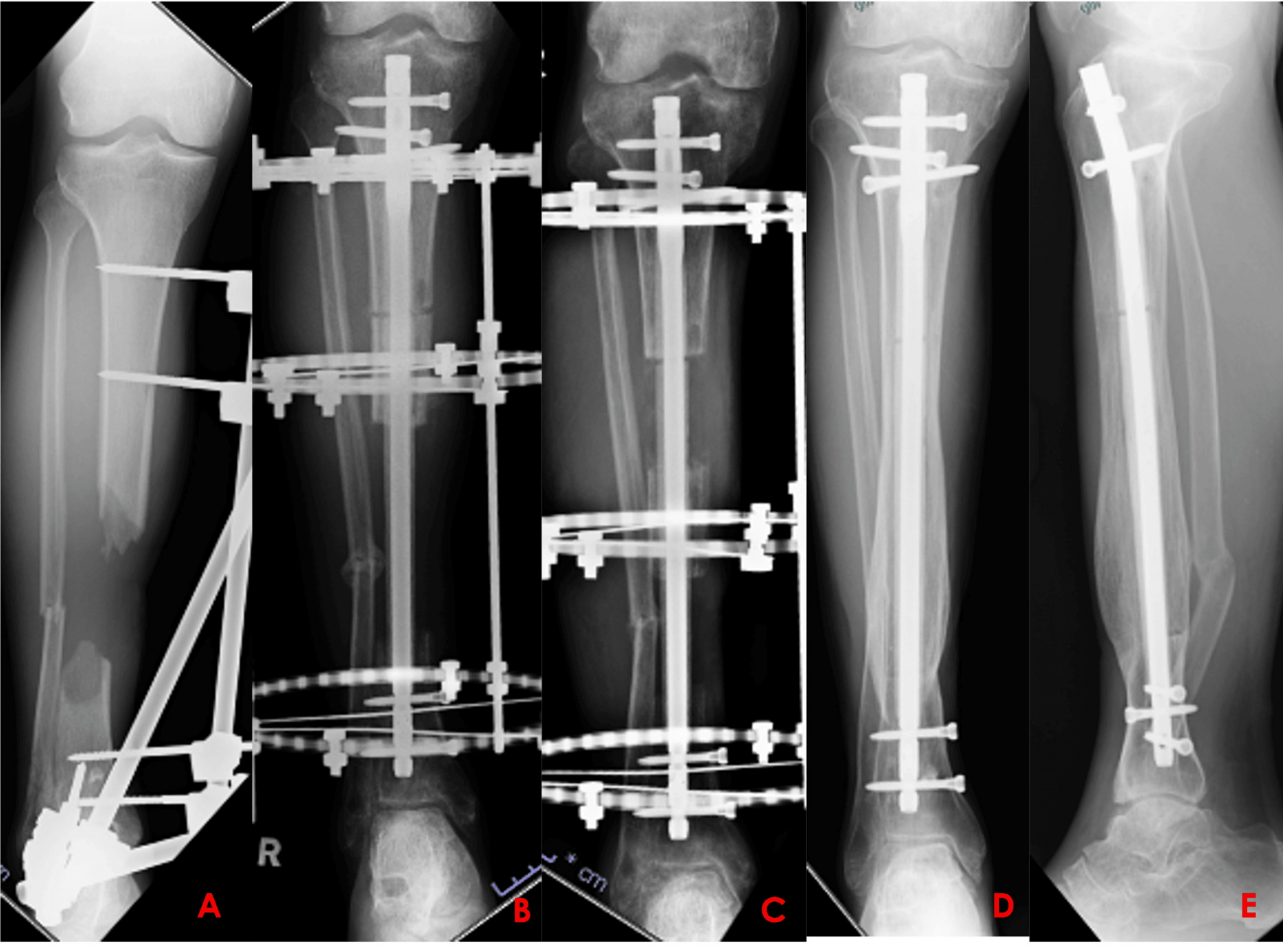
Serial radiographs of a case of bone transport over nail. (A) A trauma film shows significant bone loss of the tibia diaphysis. (B) Following an unhealthy bone resection, the defect is 10 cm. Locked intramedullary nail and ring fixator is performed with proximal corticotomy for transport over the nail. (C) Radiograph shows the bone transport segment being transported midway over the nail. (D and E) Radiographs show docking site union and transport segment consolidation at 10 months.

In case 5, the patient presented with a tibia diaphyseal defect of 10 cm. We elected to maintain the length via an antibiotic cement-coated intramedullary locked nail, and performed corticotomy and transport across the defect with the Ilizarov technique, essentially a bone transport over nail (BTON) (Figure 2). Upon completion of transport, two cycles of the accordion maneuver were performed to avoid a grafting procedure of the docking site. Once the transport regenerate was visible, the ring fixator was removed.

In case 6, the patient presented with implant failure occurring across a distal femur metaphyseal nonunion. We performed acute docking for the nonunion and replating, followed by proximal BLOP.

All patients were allowed assisted weight bearing as tolerated from the immediate postoperative period, irrespective of technique, with the aid of dedicated physiotherapists and occupational therapists. We believe that this was necessary to achieve a good functional outcome. Implant selections and fixation methods were designed to ensure sufficient stability to allow this.

Outcome measures

The primary outcome in this study was the EFI, which denotes the number of days on frame per centimeter of bone gained from distraction osteogenesis (days/cm).

Secondary outcomes included time to union, time on frame, complications, and the bone and functional outcomes as per the Association for the Study and Application of the Method of Ilizarov (ASAMI) criteria [5,6]. Union was defined radiologically as bridging callus in at least three cortices, with painless full weight bearing [3]. Complications were recorded based on the criteria established by Paley (1990) [3]. Clinic follow-ups, assessments, and recording of data were performed by the same surgical team.

Due to the small sample size (n = 6), only descriptive statistics were applied, as statistical analyses would invariably produce results that are statistically not significant. Continuous variables were reported as mean with standard deviation (SD) and range.

## Results

We recruited five males and one female, with a mean age of 32.2 ± 15.5 years. In four cases, the defect involved the femur, while in two cases, it involved the tibia. The mean defect size was 10.3 ± 3.6 cm. The mean EFI was 14 ± 3.2 days/cm. The mean TOF was 99.8 ± 30.3 days. All patients achieved union with a mean time of 8.3 ± 1.8 months. This is summarized in Table 1, and a description of the six cases is listed in Table 2.

**Table 1:** Summary of clinical outcomes (mean ± standard deviation and range).

Category	Mean ± standard deviation	Range
Age (years)	32.2 ± 15.5	14-53
Bone defect size (cm)	10.3 ± 3.6	4.5-14
External fixation index (days/cm)	14 ± 3.2	11.3-19.1
Time on frame (days)	99.8 ± 30.3	75-156
Time to union (months)	8.3 ± 1.8	6-10

**Table 2:** Data of the cohort study group describing the hybrid techniques performed for each case, and the clinical results, including the complications. F: female; M: male; TOF: time on frame; EFI: external fixation index; ASAMI: Association for the Study and Application of the Method of Ilizarov; AS: acute shortening; BLOP: bone lengthening over plate; IMT: induced membrane technique; AD: acute docking; BTON: bone transport over nail; ROM: range of motion.

Patient	Age	Sex	Defect (cm)	Technique	TOF (days)	Union (months)	EFI (days/cm)	ASAMI bone outcome	ASAMI functional outcome	Complications
Case 1	14	F	13	AS + BLOP + IMT	75	6.4	11.5	Good	Excellent	Residual varus 7°
Case 2	35	M	13	AS + BLOP + IMT	90	9	11.3	Excellent	Good	Knee ROM 0-30°
Case 3	16	M	14	AS + BLOP + IMT	80	10	11.4	Excellent	Excellent	Nil
Case 4	30	M	4.5	AD + BLOP	86	8.6	19.1	Excellent	Excellent	Early implant failure requiring revision
Case 5	45	M	10	BTON	156	10	15.6	Excellent	Excellent	Nil
Case 6	53	M	7.5	AD + BLOP	112	6	14.9	Excellent	Good	Knee ROM 15-100°

Patients were followed up two-weekly during the distraction phase to monitor for possible complications to allow for early interventions. After removal of the external fixator, patients were followed up six-weekly to monitor for consolidation. Pin tract inflammations responded well to local care and oral antibiotics, but none required unplanned removal or surgical intervention, and did not lead to any bone infections. There were no implant-related infections throughout the treatment period. One patient had an early failure of the implant, resulting in varus alignment during the consolidation phase that required surgical intervention to realign the tibia.

The ASAMI criteria are a widely accepted classification system for evaluating outcomes following distraction osteogenesis [5,6]. The bone outcomes evaluate union, infection, deformity, and leg-length discrepancy. An excellent bone result was one with union, no infection, deformity of less than 7°, and length discrepancy of less than 2.5 cm. A good result was union plus any two of the others. A fair result was the union plus one of the others. A poor result was nonunion, refracture, or none of the others. Our cases resulted in excellent bone outcomes in five patients (n = 5) and a good outcome in one patient (n = 1) with a residual varus deformity of 7°.

Functional outcomes were based on significant limp, pain, loss of 15° knee extension or ankle dorsiflexion, reflex sympathetic dystrophy, and inactivity [5,6]. An excellent result was an active individual with none of the other four criteria; a good result was an active individual with one or two of the other four criteria; and a fair result was an active individual with three or four of the other criteria or an amputation. An inactive individual was considered a poor result regardless of the other criteria. Our cases had excellent outcomes in four patients (n = 4) and good outcomes in two patients (n = 2). One had a limp and knee stiffness, which we attribute partially to a ruptured quadriceps tendon from the initial injury that was repaired. The hybrid transport with the LRS rail likely impaired the physiotherapy and range of motion recovery. Another patient had a residual fixed flexion knee deformity of 15°. All patients returned to function and daily activities.

## Discussion

Distraction osteogenesis is an established treatment in the reconstruction of large bone defects due to its versatility and reliability [2]. However, the prolonged duration of external fixation in the traditional Ilizarov technique for bone transport has been a significant limitation, often resulting in complications such as pin tract infections, joint stiffness, and poor patient compliance [3,4]. Recent advances have focused on hybrid techniques combining both internal and external fixation to reduce the EFI while maintaining the biological advantages of distraction osteogenesis [1,7]. All internal techniques, such as the IMT, have been reported to successfully reconstruct bone defects as large as 26 cm [8], but have not been consistently reproducible in our hands for large defects. Furthermore, huge amounts of autografts are required for large defects and carry significant donor site morbidity [1].

In our study, we report a mean EFI of 14 days/cm, which compares favorably to traditional Ilizarov techniques. Classic bone transport requires the use of the external fixator throughout the latency, distraction, and consolidation phase, with the expected consolidation time to be two times the distraction length [9]. As the classical technique distracts bone by 1 mm/day, this estimates the expected EFI to be 30 days/cm [9,10]. The EFI may even increase if complications occur during the course of treatment. Docking site problems following the completion of transport may also prolong the EFI due to delayed union, and may risk refracture after frame removal [1].

Strategies combining the use of internal implants have been explored, and these include bone lengthening over plate (BLOP), bone transport over plate (BTOP), and bone transport over nail (BTON) [1,7]. These methods allow earlier transition to internal stabilization, thus eliminating the need to maintain the external fixator for the consolidation phase. In addition to hybrid transport, acute docking (AD), followed by re-lengthening, allows the docking site to heal without a docking procedure [11,12]. This allows for a shorter treatment time while maintaining satisfactory outcomes. Nails preclude the ability to acutely dock and re-lengthen large defects in most cases, as that would decrease fixation stability post lengthening.

Using a nail for internal stabilization can be performed after completion of transport or used to maintain alignment and length during transport [13]. Nails are suitable for diaphyseal defects, but design limitations exclude their use in stabilizing meta-diaphyseal fractures. Nails also have the advantage of increased biomechanical stability in the lower limbs, and allow for earlier weight bearing. However, careful patient selection is required as a narrow intramedullary canal may not allow a smooth bone transport over a nail. In BTON, it is necessary to maintain the external fixator until consolidation of the distraction callus is observed. The external fixator may be removed earlier if additional procedures are taken to stabilize the docking site [1]. These include plate fixations of the docking site or locking bolts inserted into predrilled holes in the nail. In our series, one case utilized a nail for BTON. The EFI of 15.6 days/cm was slightly longer as we elected not to perform a docking procedure. The accordion technique was used to compress the docking site, and the frame was kept longer for docking site union and distraction callus consolidation [14].

Conversely, peri-articular locking plates allow for stable fixation nearer to joints, as observed in our cohort of distal femur fractures involving extensive meta-diaphyseal bone loss. Plate fixation is more versatile, such that it can be applied to fractures where intramedullary nailing would not confer adequate biomechanical stability. Additionally, using a plate for hybrid transport precludes the risks of a narrow canal. Plates allow for compression across a docking site fixation during acute docking. Following transport, the distraction callus can be easily stabilized with percutaneous screw insertion of the locking plate to the mobile segment. This allows for earlier removal of the external fixator for the consolidation phase [1]. Our cohort of BLOP patients had a shorter EFI of 11.3 to 11.5 days/cm, effectively a reduction of two-thirds of the EFI of a traditional Ilizarov transport.

The combination of IMT with distraction osteogenesis reflects an evolving strategy in the reconstruction of large bone defects. In our series, the incorporation of the IMT in defects exceeding 10 cm further contributed to reducing transport distance and overall time on frame. This combined approach allows partial defect management through biological reconstruction while reducing the amount of distraction required. Although some authors demonstrated no clear association between defect size and union rates, the IMT has conventionally been recommended for smaller defects [8,15]. This has been more consistent with our local practice and clinical results. Furthermore, by reducing the fracture gap, a smaller amount of harvested autograft was required for the second-stage IMT.

Our cohort patients did not experience pin tract infections requiring unplanned removal or revisions. That was partly attributed to patient selection. Our patients were able to perform daily pin tract care independently. Throughout the distraction phase, they were closely monitored for possible complications in order to intervene early, as hybrid transport carries a risk of seeding infection to the internal implant from the external fixator. All patients achieved union without implant-related infections. This suggests that in a controlled environment, the hybrid transport does not necessarily increase the infection risk when appropriate protocols are carefully adhered to. The shorter time on frame likely afforded better patient tolerance of the external fixator and improved compliance and overall experience. This is relevant in the modern-day practice as patient-centered outcomes are likely to play an important role in treatment selection [16].

One patient had an early implant failure during the consolidation phase that resulted in a varus deformity of the tibia. This required surgical intervention to realign the tibia. This highlights the need for close monitoring to intervene early. Two patients had knee joint stiffness, a complication recognized in the existing literature from the use of distraction osteogenesis [3]. Despite this, both were active and independent. This is likely attributed to the shorter time on frame as opposed to the prolonged external fixator of the classical transport technique. Our first case of acute shortening and re-lengthening via BLOP had a residual varus deformity of 7°. Meticulous preoperative planning to account for re-lengthening along the mechanical axis is necessary to prevent residual coronal plane deformities. Subsequent cases of BLOP did not have the same problem.

Bone and functional outcomes assessed with the ASAMI criteria were favorable, with all patients achieving good to excellent results. The ASAMI classification remains one of the most widely used systems for evaluating outcomes after limb reconstruction via distraction osteogenesis and allows for meaningful comparison with existing literature [5,6].

Limitations

This study was limited by its small sample size and retrospective design. The heterogeneity of techniques reflects the individualized nature of limb reconstruction, but makes direct comparisons between techniques difficult. The outcomes were assessed retrospectively, which may introduce bias. The absence of a control group limits cross-comparisons and analysis to the traditional Ilizarov technique.

Future studies comparing hybrid transport techniques with all internal motorized intramedullary lengthening systems are warranted, but cost and availability remain a concern. Although modern technology has made such techniques possible, they are not without their device-related complications.

## Conclusions

Hybrid bone transport techniques combining internal implants with established external reconstruction methods reduce the external fixator index and overall time on frame compared to classical bone transport while maintaining excellent union rates and favorable functional outcomes. The addition of the induced membrane technique to the reconstruction protocol further reduced the time on frame and the distance of bone transported. There was no increased risk of cross-infection in a controlled environment with strict adherence to care protocols. Although promising, this study was limited by a small sample size. Further validation of hybrid bone transport benefits warrants a larger-scale, prospective study employing standardized surgical techniques to minimize procedural heterogeneity.
